# Comparison of Hook and Straight Steel Fibers Addition on Malaysian Fly Ash-Based Geopolymer Concrete on the Slump, Density, Water Absorption and Mechanical Properties

**DOI:** 10.3390/ma14051310

**Published:** 2021-03-09

**Authors:** Meor Ahmad Faris, Mohd Mustafa Al Bakri Abdullah, Ratnasamy Muniandy, Mohammad Firdaus Abu Hashim, Katarzyna Błoch, Bartłomiej Jeż, Sebastian Garus, Paweł Palutkiewicz, Nurul Aida Mohd Mortar, Mohd Fathullah Ghazali

**Affiliations:** 1 Faculty of Mechanical Engineering Technology, University Malaysia Perlis (UniMAP), Arau 02600, Perlis, Malaysia; firdaushashim@unimap.edu.my (M.F.A.H.); fathullah@unimap.edu.my (M.F.G.); 2 Center of Excellence Geopolymer and Green Technology (CEGeoGTech), Universiti Malaysia Perlis (UniMAP), Kangar 01000, Perlis, Malaysia; nurulaida@unimap.edu.my; 3 Faculty of Chemical Engineering Technology, University Malaysia Perlis (UniMAP), Arau 02600, Perlis, Malaysia; 4 Department of Civil Engineering, University Putra Malaysia, Serdang 43400, Selangor, Malaysia; ratnas@eng.upm.edu.my; 5 Department of Physics, Częstochowa University of Technology, 42-200 Częstochowa, Poland; katarzyna.bloch@pcz.pl (K.B.); bartek199.91@o2.pl (B.J.); 6 Faculty of Mechanical Engineering and Computer Science, Częstochowa University of Technology, 42-200 Częstochowa, Poland; gari.sg@gmail.com (S.G.); palutkiewicz@ipp.pcz.pl (P.P.)

**Keywords:** geopolymer, alkali activated material, reinforced concrete, steel fiber, physical properties, mechanical properties, flexural

## Abstract

Geopolymer concrete has the potential to replace ordinary Portland cement which can reduce carbon dioxide emission to the environment. The addition of different amounts of steel fibers, as well as different types of end-shape fibers, could alter the performance of geopolymer concrete. The source of aluminosilicate (fly ash) used in the production of geopolymer concrete may lead to a different result. This study focuses on the comparison between Malaysian fly ash geopolymer concrete with the addition of hooked steel fibers and geopolymer concrete with the addition of straight-end steel fibers to the physical and mechanical properties. Malaysian fly ash was first characterized by X-ray fluorescence (XRF) to identify the chemical composition. The sample of steel fiber reinforced geopolymer concrete was produced by mixing fly ash, alkali activators, aggregates, and specific amounts of hook or straight steel fibers. The steel fibers addition for both types of fibers are 0%, 0.5%, 1.0%, 1.5%, and 2.0% by volume percentage. The samples were cured at room temperature. The physical properties (slump, density, and water absorption) of reinforced geopolymer concrete were studied. Meanwhile, a mechanical performance which is compressive, as well as the flexural strength was studied. The results show that the pattern in physical properties of geopolymer concrete for both types of fibers addition is almost similar where the slump is decreased with density and water absorption is increased with the increasing amount of fibers addition. However, the addition of hook steel fiber to the geopolymer concrete produced a lower slump than the addition of straight steel fibers. Meanwhile, the addition of hook steel fiber to the geopolymer concrete shows a higher density and water absorption compared to the sample with the addition of straight steel fibers. However, the difference is not significant. Besides, samples with the addition of hook steel fibers give better performance for compressive and flexural strength compared to the samples with the addition of straight steel fibers where the highest is at 1.0% of fibers addition.

## 1. Introduction

Currently, geopolymer concrete has emerged in the last two decades as new engineering materials with the potential of becoming a significant element in the environmentally sustainable construction and construction industry [1]. The reaction of aluminosilicate—containing fly ash with alkali produces an inorganic polymer binder, which is called geopolymer. A concrete geopolymer was studied by several researchers [2,3,4,5]. Geopolymer was also developed for other applications including lightweight concrete [6], material for computer numerical control (CNC) cutting tools [7,8,9,10], piping materials [11], and water treatment [12].

Based on previous research, the interfacial bonding carried out using the pull-out test have shown that Ordinary Portland Cement (OPC) mortars are generally excellent [13]. Recently, the works related to steel reinforcing fiber on Ordinary Portland Cement (OPC) composite, including fly ash, rice hush-bark, and silica fume have been studied. The results indicated that the OPC composite bonding strength was improved on the aging of the 28th day [14].

Hooked steel fiber is widely used for OPC concrete reinforcement [15,16,17]. The material properties of fibers are usually more dominant in affecting the performance of a reinforced geopolymer composite than binders [18]. The role of the fiber is very important as it is mainly reflected when the concrete starts to crack and it also improves the post-cracking performance as there is the fiber bridging of the crack section. It is said that adding steel fibers to concrete can produce a better crack control effect and improve the tensile strength before and after cracking initiates [19,20,21]. The inclusion of steel fibers could help to improve fatigue strength and dynamic resistance of the concrete. However, as the amount of steel fiber added increases, the workability of concrete will decrease [21].

Concrete geopolymer has excellent strength without any fiber reinforcement [1,22] and good fire resistance. Fibers are incorporated into OPC concrete to overcome this weakness, producing materials with increased tensile strength, ductility, toughness, and increased durability properties. Fiber efficiency in the concrete mixture depends on factors such as fiber matrix properties, the volume of fiber inclusion, fiber geometry, the types of fiber, as well as fiber orientation. To improve durability, strength, and different properties of hardened concrete, a wide range of fibers must be studied. Most of the fibers are made of steel, carbon, or polymer [23]. The selection of steel fiber is chosen for this research to gather the data of strength, and different properties on geopolymer concrete.

Research on the addition of steel fibers in OPC concrete to enhance the performance has been extensively studied by many researchers [24,25,26] over the world but is quite limited to fly ash-based geopolymer concrete. In the production of geopolymer concrete, the chemical composition of raw material, which is fly ash, is not standardized, especially as it comes from different sources. The different chemical content of fly ash may influence to the different properties and performance of geopolymer concrete. Besides, the study on mechanical and physical properties of fly ash-based geopolymer concrete with the reinforcement of steel fibers is limited. This paper aims to study the effects of steel fibers as an addition to the physical and mechanical properties of Malaysian fly ash-based geopolymer concrete.

## 2. Materials and Methodology

### 2.1. Preparation of SFRGC

Steel Fiber Reinforcement Geopolymer Concrete (SFRGC) is mixing between geopolymer binder which is fly ash, an alkali activator, aggregates, and a specific amount of steel fibers. Firstly, an alkali activator was produced by mixing sodium silicate (Na_2_SiO_3_) and sodium hydroxide (NaOH) at a ratio equal to 2.5. The Na_2_SiO_3_ was from South Pacific Chemicals Industry Sdn. Bhd. (SPCI), Penang, Malaysia with a ratio of SiO and Na_2_O equal to 3.2. NaOH is in a form of a pallet that was bought from Farmosa Plastic Corporation, Kaohsiung, Taiwan. The NaOH pellet was first diluted in distilled water and as a result, a concentration of 12 M was produced.

**Figure 1:**
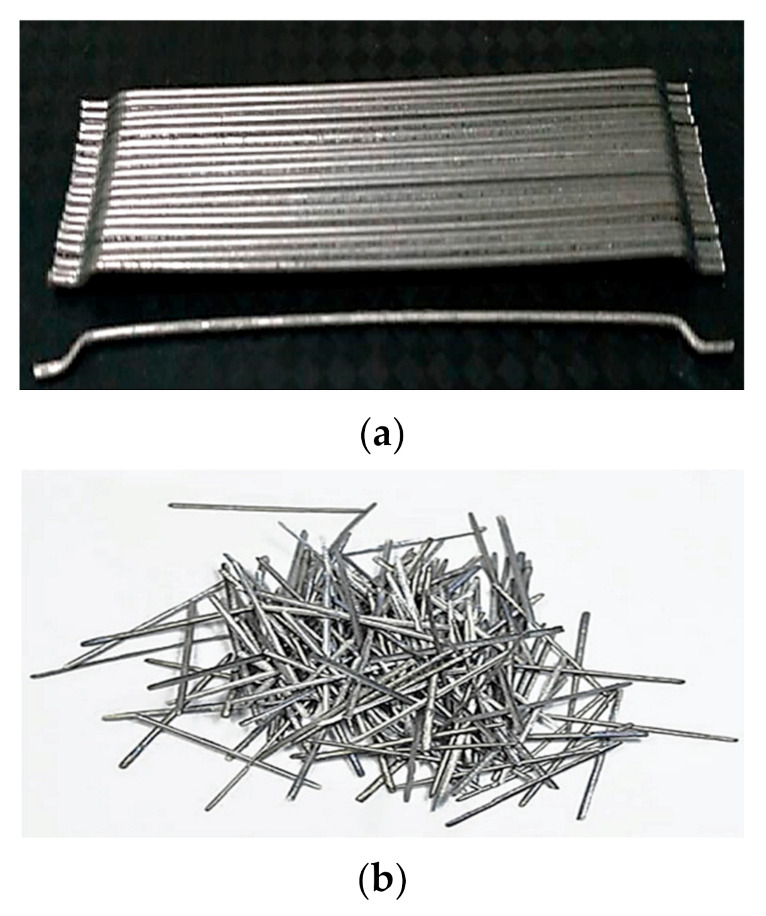
Steel fibers: (**a**) Hook fibers and (**b**) straight fibers.

Fly ash was taken from the electric power plant Manjung, Perak, Malaysia. Aggregates, which are river sand and granite, were used at a maximum size equal to 4.75 mm and 20 mm respectively. The ratio of fly ash to alkali activators is equal to 2. The steel fibers used in this experiment are hook and straight end as shown in Figure 1. The percentage of steel fibers as an addition in the geopolymer concrete mixture has been set based on the volume fraction of samples which are 0%, 0.5%, 1.0%, 1.5%, and 2.0%. The details mix proportion of the SFRGC is tabulated in Table 1. Besides, the details of the end-shape steel fibers specification used in the production of SFRGC was summarized in Table 2 as hook steel fiber and in Table 3 as straight steel fiber.

The samples of SFRGC were cast in a mold size of 100 mm × 100 mm × 100 mm for physical (workability, density, and water absorption) and mechanical (compressive and flexural) tests. Samples were taken out from the mold after 24 h and cured at room temperature for 28 days.

### 2.2. Slump Test

Workability of SGRGC was measured by a slump test. The slump test was performed based on ASTM C143 [27]. After the mixing process, the fresh geopolymer concrete was poured into a slump cone in three layers. Each layer was compacted by rodding 25 strokes by suing the tamping rod. The excess of fresh SFRGC was removed by scrapping off from the top of the cone. The slump cone then was immediately removed from the fresh SFRGC by raising it up vertically. The slump was measured by determining the distance between the top of the slump cone and the displaced original center of the top surface of the fresh SFRGC.

### 2.3. Density Test

A density test was conducted after the sample solidified for 28 days. A sample was immersed in water for 24 h at ambient temperature. The sample of SFRGC was placed separately (no contact) from each other in a water tank. The top of the sample surface was not lower than 150 mm below the water surface. The immersed sample was placed on a wire mesh to ensure there is a gap between the sample and bottom of the water container at a distance of about 3 mm.

The sample of SFRGC that completely was immersed in water for 24 h was weighed and recorded as immersed weight (Wi). The sample then was removed from the water tank and allowed to dry for 1 min. Any visible water on the surface of the sample was removed by a damp cloth. Then, the sample was weighed and recorded as saturated weighed (Ws). After that, the sample was dried in an oven at a temperature of 110 °C for 24 h. Then, the dried sample was weighed and labeled as dried weight (Wd). The detail calculation of density of SFRGC is summarized as in Equation 1:

(1) $$\mathrm{Density},\;D=\left(\frac{W_{d}}{W_{s}-W_{i}}\right)\;\times \;1000$$

where:

$W_{d}$ = oven dry weight of specimen (kg);

$W_{s}$ = saturated weight of specimen (kg);

$W_{i}$ = immersed weight of specimen (kg).

### 2.4. Water Absorption Test

Meanwhile, a water absorption test was conducted to determine the moisture content of the samples. The experimental procedure of water absorption was conducted in a similar way to the density test in Section 2.2 but different with the calculation. The water absorption of the SFRGC was measured by Equation (2):

(2) $$\mathrm{Percentage}\;\mathrm{of}\;\mathrm{water}\;\mathrm{absorption}\;\left(\%\right)=\left(\frac{W_{s}-{\;W}_{d}}{W_{d}}\right)\;\times \;100$$

where:

$W_{d}$ = oven dry weight of specimen (kg);

$W_{s}$ = saturated weight of specimen (kg);

$W_{i}$ = immersed weight of specimen (kg).

### 2.5. Compression Test

The compressive strength of sample SFRGC was measured by following standard BS 1881-116 [28] using Universal Testing Machine (UTM) Automatic Max (Instron, 5569, Norwood, MA, USA). This testing was conducted for sample cured at 28 days in ambient temperature. The load speed was adjusted to 0.1 kN/s.

### 2.6. Flexural Test

The flexural test, was performed to measure the flexural strength of the sample. The sample was subjected to 4 point bending and tested by using the UTM model Automatic Max (Instron, 5569, Norwood, MA, USA). The testing was conducted by following ASTM C1018 [29]. The constant deflection rate applied in this testing was in the range of 0.05–0.10 mm/min. The lower and upper support are 300 mm and 100 mm respectively. The sample was tested at 28 days after being cured at ambient temperature.

## 3. Results and Discussion

### 3.1. Chemical Composition

The chemical composition of fly ash that has been used in the production of geopolymer concrete for both types of fibers is summarized in Table 4. The total amounts of SiO_2_ and Al_2_O_3_ are 53.5% from the total composition. Fly ash used in this experiment fulfils the fundamental as a source of material to be used as a precursor in a geopolymerization process where it is rich in Si and Al, which will be activated by alkali solution. This fly ash is verified as Class F by ASTM C618 [30]. This verification is based on the chemical content of CaO, Al_2_O_3_, SiO_2_, and Fe_2_O_3_.

### 3.2. Slump

**Figure 2:**
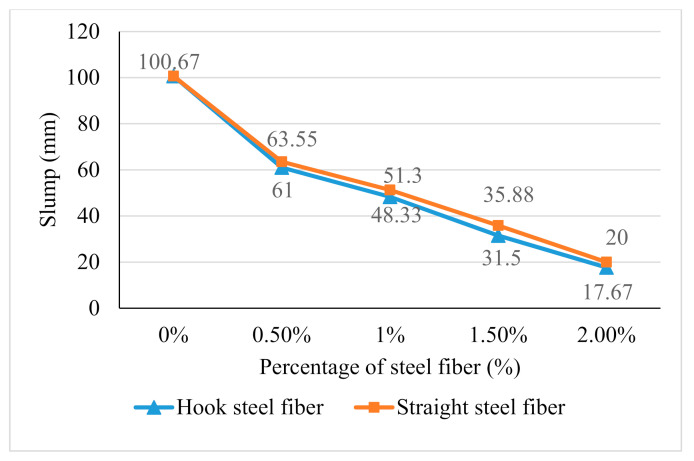
Slump of geopolymer concrete with the addition of hook steel fibers and straight steel fibers.

The slump was measured by using a conventional slump cone. The slump test in this research was measured to find consistency and workability of geopolymer concrete between hook steel fiber and straight steel fiber reinforced. Figure 2 showed the decrease trend workability between hook steel fiber and straight steel fiber.

**Figure 3:**
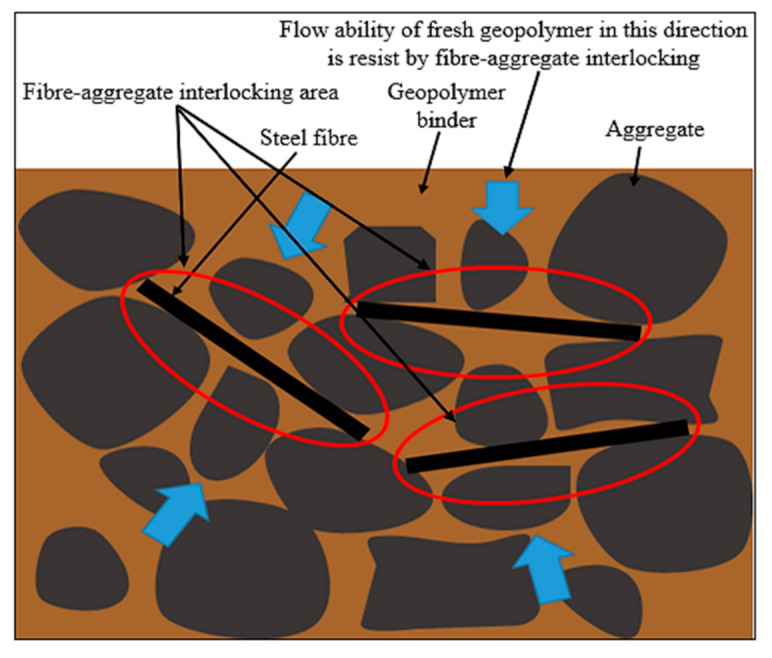
Flow of fresh reinforced geopolymer concrete is resisted by fiber-aggregate interlocking.

Figure 2 illustrates that the slump test result of geopolymer concrete without the addition of steel fibers was 100.67 mm. The slump of geopolymer concrete for hook steel fibers addition decreased with the increasing additions of steel fibers from 0% to 2% which are 61 mm (0.5% steel fibers), 48.33 mm (1% steel fibers), 31.5 mm (1.5% steel fibers), and 17.67 mm (2.0% steel fiber). Meanwhile, the slump of straight steel fiber had also decreased with the increasing addition of steel fibers which is 63.55 mm (0.5% steel fibers), 51.3 mm (1% steel fibers), 35.88 mm (1.5% steel fibers), and 20 mm (2.0% steel fiber). This has shown that the existence of steel fibers will reduce the workability of geopolymer concrete. The workability of concrete will significantly decrease due to the increase of resistance between the steel fibers and geopolymer matrix called interlocking as shown in Figure 3.

Meanwhile, the slump of hook steel fiber was lower than the straight steel fiber due to the function of the hook end that creates higher flow resistance to the fresh geopolymer concrete compared to the geopolymer concrete reinforced with straight steel fiber. The medium value for the slump test of OPC concrete ranges between 50 mm to 100 mm. Based on this result, it can be concluded that the range of 17.67 mm to 100 mm can be considered as low and medium workability. This indicates that the mixing process involved with these materials is suitable to be produced by using a vibrator or manually.

### 3.3. Density

**Figure 4:**
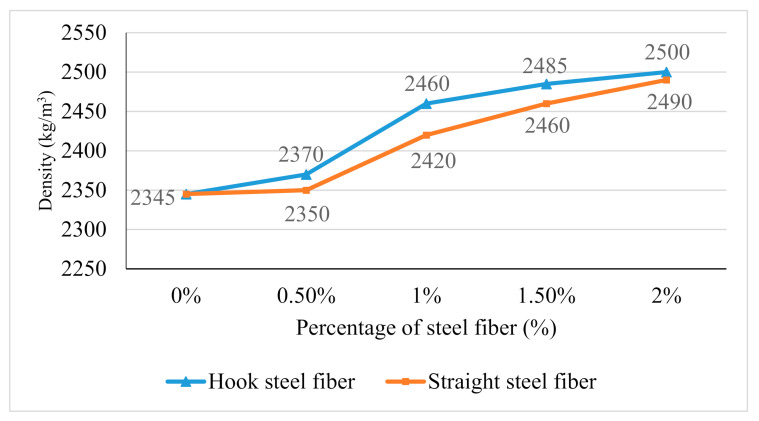
Density of hook and straight steel fiber against steel fibers addition.

The density of a substance is measured as mass per unit volume. All the samples were measured after being cured for 28 days at room temperature by their mass and divided by the size of mold which is 100 mm × 100 mm × 100 mm. Figure 4 shows the graph comparison of density between hooked steel fiber and straight steel with different fibers addition.

From the results, the density of 2% steel fiber additions for both hooked and straight steel fiber was the highest with 0.25 kg/m^3^ and 0.249 kg/m^3^ respectively. The density of geopolymer increased proportionally with the increase of both steel fibers addition. The density of geopolymer concrete with the addition of hook steel fibers seems higher than the density of geopolymer concrete with the addition of straight steel fibers in all percentage of fibers addition. The addition of steel fiber increased the density because of different specific gravity for steel fiber which is 7800 kg/m^3^ compared to a normal geopolymer which is 2345 kg/m^3^ and it gives an impact for a density value of SFRGC. The increase of concrete density by the addition of aggregate or reinforcement fibers with higher specific gravity was also mentioned by Harshavardhan and Bala Murugan [31].

### 3.4. Water Absorption

**Figure 5:**
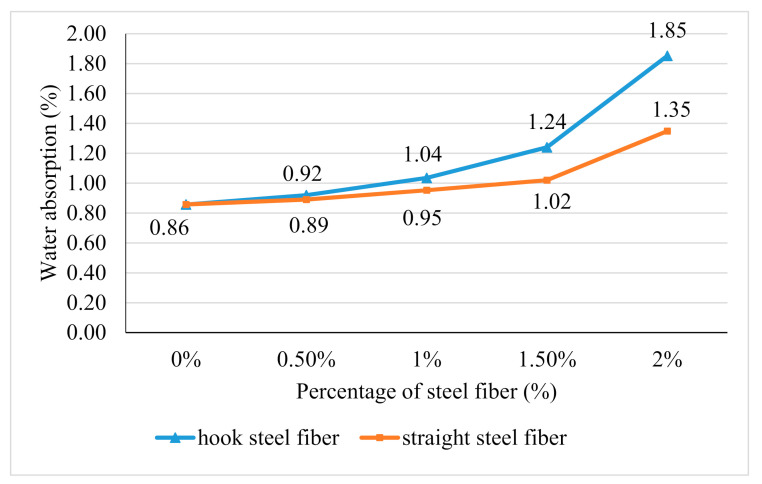
Water absorption of geopolymer concrete with the addition of hooked and straight steel fibers.

Figure 5 shows the result of the water absorption test. The water absorption of geopolymer concrete was increased with the increase of both types of fiber additions. The highest water absorption for geopolymer concrete with the addition of hook steel fibers and straight steel fibers are 1.35% and 1.85% which is 2% of the steel fibers addition. This is due to the workability which decreased with the addition of steel fibers obtained in this research, which can lead to an increase in pores formation. The water absorption of geopolymer concrete with the addition of hook steel fibers seems higher than the straight steel fiber. This is due to the workability of geopolymer concrete with the addition of hook steel fibers which is lower compared to the geopolymer concrete with the addition of straight steel fibers.

### 3.5. Compressive Strength

**Figure 6:**
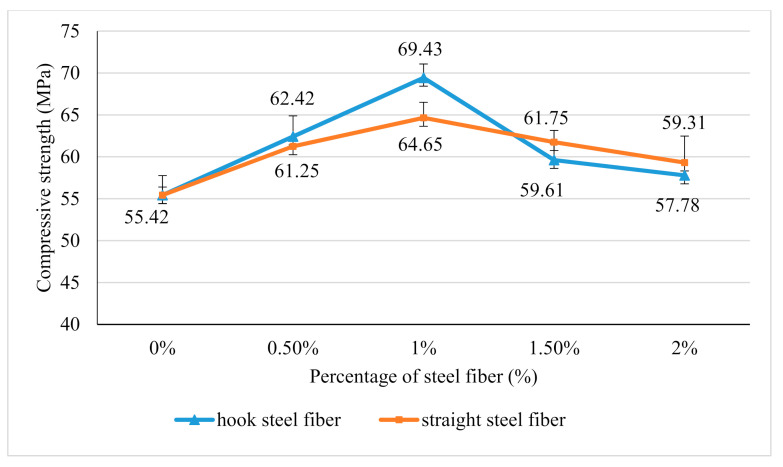
Compressive strength of hook steel fiber and straight steel fiber reinforced geopolymer concrete versus steel fibers addition.

The compressive strength of steel fiber reinforced geopolymer concrete for both samples are summarized as in Figure 6. The addition of hook steel fibers and straight steel fibers seems to improve the compressive strength of geopolymer concrete until its maximum value at 1% of fibers addition. This is due to the steel fibers that help to stop the propagation of crack during the compression load. Besides, the compressive strength of hook steel fibers addition seems better than the straight steel fibers addition. This is due to the contribution of the end types of steel fibers in which the anchorage of hook steel fibers will produce higher resistance when slipping out from the geopolymer binder during the cracking compared to the straight steel fibers that do not have anchorage.

However, the compressive strength of geopolymer concrete decreases as the addition of the fibers exceeds 1.5%. This may be due to the poor workability of geopolymer concrete with the addition of steel fibers starting at 1.5%, which is around 31.5 mm and 35.88 mm for the inclusion of hook and straight steel fiber respectively.

### 3.6. Flexural Strength

**Figure 7:**
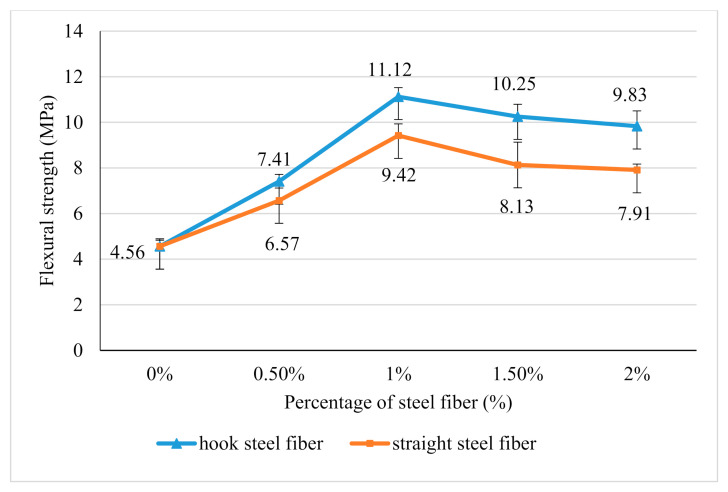
Flexural strength of hook steel fiber and straight steel fiber reinforced geopolymer concrete versus steel fibers addition.

**Figure 8:**
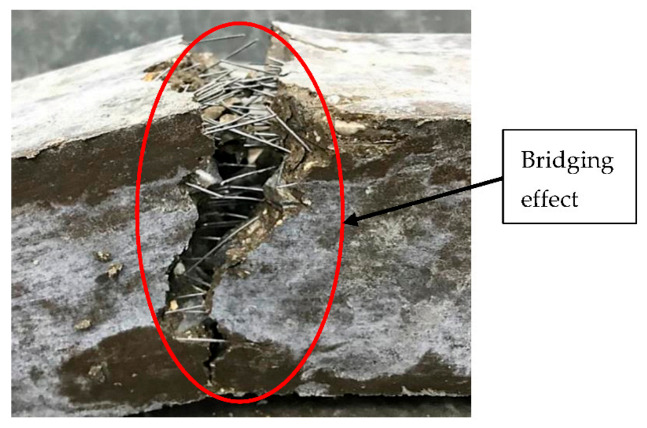
Sample of Steel Fiber Reinforcement Geopolymer Concrete (SFRGC) after a flexural test shows the bridging effect at the cracking area.

The effect of steel fibers addition to the flexural strength of geopolymer concrete is summarized in Figure 7. The inclusion of hook steel fibers and straight steel fibers increases the flexural strength of geopolymer concrete until the addition of a maximum fiber at 1.5% which is 11.12 MPa and 9.42 MPa respectively. The improvement of flexural strength is due to the steel fibers, which functioned to stop the propagation of crack during the flexural testing by creating a bridging at the crack area as shown in Figure 8. The higher the amount of steel fibers, the higher the flexural load that the sample can absorb before completely failing.

However, the performance of flexural drops when the steel fibers addition exceeds 1.5% for both types of fibers. This is related to the poor workability of samples when steel fibers are added at a high amount. This poor workability may affect the steel fibers dispersion resulting in the steel fibers not being dispersed uniformly. Hence, the absorption capacity inside the sample is imbalance when loading is applied. Therefore, the sample tends to crack at the area that contains fewer fibers.

Besides, the addition of hooked steel fibers shows a higher flexural strength compared to the straight steel fibers at all percentage of steel fibers addition. This is due to the anchorage of hooked steel fibers, which provides extra loading capacities during the flexural test compared to the straight steel fibers. The anchorage of hooked end steel fibers increases the resistance and friction between the fibers and geopolymer matrix when a load is applied. This indicates that higher energy is required to slip out the hooked end steel fibers from the geopolymer matrix.

## 4. Conclusions

The reinforcement of Malaysian fly ash-based geopolymer concrete by the addition of hook and straight steel fibers showed some influence on the physical and mechanical properties which are slump, density, water absorption, compressive, and flexural strength. Overall, the changes of the slump, density, and water absorption for geopolymer concrete with reinforcement of hook and straight steel fibers were not significant and is still acceptable to be used as concrete in normal applications. However, there was a significant improvement for both types of samples in the mechanical properties especially the flexural strength which is due to the bridging effect. The addition of hook steel fibers has proven to give a higher improvement to mechanical properties compared to the addition of straight steel fibers. Overall, the types of hooked-end steel fibers are better to be used as reinforcement on Malaysian fly ash-based geopolymer concrete compared to straight steel fibers due to its better mechanical performance with acceptable physical properties. The source of alumino-silicate, which is the Malaysian fly ash, has proven to be used as a precursor in the production of steel fiber reinforced geopolymer concrete.

## Figures and Tables

**Table 1:** Mix design of steel fiber reinforced geopolymer concrete.

Steel Fibers Addition (%)	Fly Ash (kg/m^3^)	Coarse Aggregate (kg/m^3^)	Fine Aggregate (kg/m^3^)	Sodium Silicate (kg/m^3^)	Sodium Hydroxide (kg/m^3^)
0	640	864	576	229	91
0.5	630.44	851.10	567.40	225.58	89.42
1.0	620.99	838.34	558.89	222.20	88.30
1.5	611.63	825.70	550.47	218.85	86.97
2.0	602.36	813.19	542.13	215.53	85.65

**Table 2:** Specifications of hooked steel fibers.

Items	Specifications
Fiber type	Hooked
Diameter (mm)	0.60
Length (mm)	0.75
Aspect ratio	80

**Table 3:** Specifications of straight steel fibers.

Items	Specifications
Fiber type	Straight
Diameter (mm)	0.60
Length (mm)	0.75
Aspect ratio	80

**Table 4:** Chemical composition of fly ash.

Element	Percentage (%)
SiO_2_	38.80
Fe_2_O_3_	19.48
CaO	18.10
Al_2_O_3_	14.70
MgO	3.30
K_2_O	1.79
SO_3_	1.50
MnO	0.16
BaO	0.27
TiO_2_	1.02

## Data Availability

The data presented in this study are available on request from the corresponding author.
